# Influence of the type of reciprocating motion on the cyclic fatigue resistance of reciprocating files in a dynamic model

**DOI:** 10.1186/s12903-021-01538-8

**Published:** 2021-04-07

**Authors:** Álvaro Zubizarreta-Macho, Alberto Albaladejo Martínez, Carlos Falcão Costa, Norberto Quispe-López, Ruben Agustín-Panadero, Jesús Mena-Álvarez

**Affiliations:** 1 Department of Endodontics, Faculty of Dentistry, Alfonso X el Sabio University, 28691 Madrid, Spain; 2 Department of Dentistry, School of Medicine, University of Salamanca, 37008 Salamanca, Spain; 3 Faculty of Health Sciences, Fernando Pessoa University, 4150-518 Porto, Portugal; 4 Department of Stomatology, Faculty of Medicine and Dentistry, University of Valencia, 46010 Valencia, Spain; 5 Department of Endodontics, Faculty of Dentistry, Alfonso X El Sabio University, Albarracin 35, 28037 Madrid, Spain

**Keywords:** Endodontics, Cyclic fatigue, Nitinol, Reciprocating movement

## Abstract

**Background:**

The aim of this study was to compare the influence of two novel reciprocating movements on the cyclic fatigue resistance of endodontic reciprocating files.

**Methods:**

30 Procodile® (Komet Medical, Lemgo, Germany) files were selected in this study and distributed according to the following study groups depending on the movements to be performed: ReFlex Dynamic (*n* = 10), ReFlex Smart (*n* = 10) and Reciproc (*n* = 10) reciprocating movement. These files were fixed to a specific dynamic cyclic fatigue device designed and manufactured by 3D impression to simulate the pecking motion performed by the operator. The time to failure and the number of cycles of in-and-out of the endodontic files was registered. The results were analyzed by ANOVA and Weibull statistics.

**Results:**

Statistically significant differences were found when the number of cycles of in-and-out movement and the time to failure of ReFlex Dynamic and Reciproc reciprocating movement (*p* < 0.001) and between ReFlex Smart and Reciproc reciprocating movement (*p* < 0.001) were compared in pairs. However, no statistically significant differences were observed between time to failure and number of cycles of in-and-out movement of ReFlex Dynamic and ReFlex Smart reciprocating movement (*p* = 0.253).

**Conclusions:**

The ReFlex Smart reciprocating movement increased the cyclic fatigue resistance of endodontic reciprocating files compared with traditional reciprocating movement.

## Background

Nickel–titanium (NiTi) rotary files have improved accuracy, reduced apical foramen transportation and reduced working time, with respect to traditional stainless steel endodontic files [1]. However, the failure of NiTi rotary files is still a complication related to endodontic rotary instruments difficult to solve [2]. Failure of NiTi rotary files can occur by cyclic bending fatigue or torsional overload [3]. Torsional overload occurs when the tip of the file is locked inside the root canal system. Cyclic bending fatigue is caused when the file is submitted to alternating compressive and tensile stress cycles at the maximum curvature of the curved root canal [4]. The arrival of reciprocating single-file systems with speed- and torque-controlled motor systems has had a great impact on the field of endodontics. Single-file systems have demonstrated the ability to clean and shape the root canal system with fewer instruments, which implies a reduced working time. In addition, they have shown a negative effect on postoperative pain after root canal treatment [5], a high capability to maintain the original canal anatomy without removing excess dentin and enhancing a more centered preparation compared with rotary multiple-file systems [6]; these files have a short learning curve [7] although they do not show statistically significant differences regarding their antibacterial efficacy compared to rotary multiple-file systems [8]. However, single-file systems are submitted to high levels of cyclic and torsional fatigue, which might lead to fracturing of reciprocating files [9]. The reciprocating movement associated with single-file systems has been shown to extend the lifetime of NiTi rotary files compared with continuous rotation, thus increasing the cyclic fatigue resistance of reciprocating files [8]. The previously described Wave One and Reciproc reciprocating movement instruments perform wide and continuously counter clockwise (CCW) (170° and 150°, respectively) movement and reduce the fatigue of the reciprocating files by moving in a smaller angle in the clockwise (CW) direction (50° and 30°, respectively) [10, 11,]. The large CCW reciprocating angle allows the reciprocating file to cut the root canal dentine and advance in the root canal system, whereas the smaller CW reciprocating angle allows the reciprocating file to disengage from the dentine to reduce the screwing effect and file breakage. However, the ReFlex Dynamic reciprocating movement performed by EndoPilot® endodontic handpiece (Komet Medical, Lemgo, Germany) starts moving the reciprocating files (Procodile®, Komet Medical, Lemgo, Germany) with a widely CCW movement, but stops and continues moving CCW without turning in reverse if no resistance of the files is detected by the handpiece management software. However, if the handpiece management software detects resistance of the reciprocating files, EndoPilot® is able to change the movement of the fatigued files in the reverse direction (CW). The ReFlex Smart reciprocating movement performed by EndoPilot® handpiece starts making the same movement as the ReFlex Dynamic reciprocating movement, but it is also able to turn twice in the reverse direction (CW) if the handpiece management software detects resistance on the fatigued files.

Taking those facts into account, the aim of this work was to analyze and compare the influence of two novel reciprocating movements on the cyclic fatigue resistance of endodontic reciprocating files, with a null hypothesis (H0) stating that there would be no difference between the reciprocating movements with regard to the cyclic fatigue resistance. The incorporation of a new movement and a new design in the endodontic file justifies this study. It should be noted that Procodile file is made of a conventional NiTi alloy.

## Methods

### Study design

Procodile® (Komet Medical, Lemgo, Germany) is a NiTi endodontic reciprocating file with one-file system. Procodile has a double-S cross-section, variable tapered core, 0.25 mm tip diameter, 6% continous taper, 25 mm in length and CCW reciprocating motion. A sample of 30 new files were utilized in this in vitro study. Previously to use, all endodontic files were inspected under a stereomicroscope (SZR-10, Optika, Bergamo, Italy) to observe possible defects and none were discarded. The endodontic files were randomized (Epidat 4.1, Galicia, Spain) and distributed into the following study groups: A: ReFlex Dynamic reciprocating movement (n = 10); B: ReFlex Smart reciprocating movement (n = 10); and C: Reciproc reciprocating movement (n = 10).

### Dynamic cyclic fatigue test device

To perform the dynamic cyclic fatigue tests was used a custom-made device (utility model patent number ES1219520) previously described [12]. The structure of experimental cyclic fatigue model was designed by computer aided design/computer aided engineering (CAD/CAE) 2D/3D software (Midas FX+®, Brunleys, Milton Keynes, UK) and manufactured by 3D impression (ProJet® 6000 3D Systems^©^, Rock Hill, SC, USA).

**Fig. 1:**
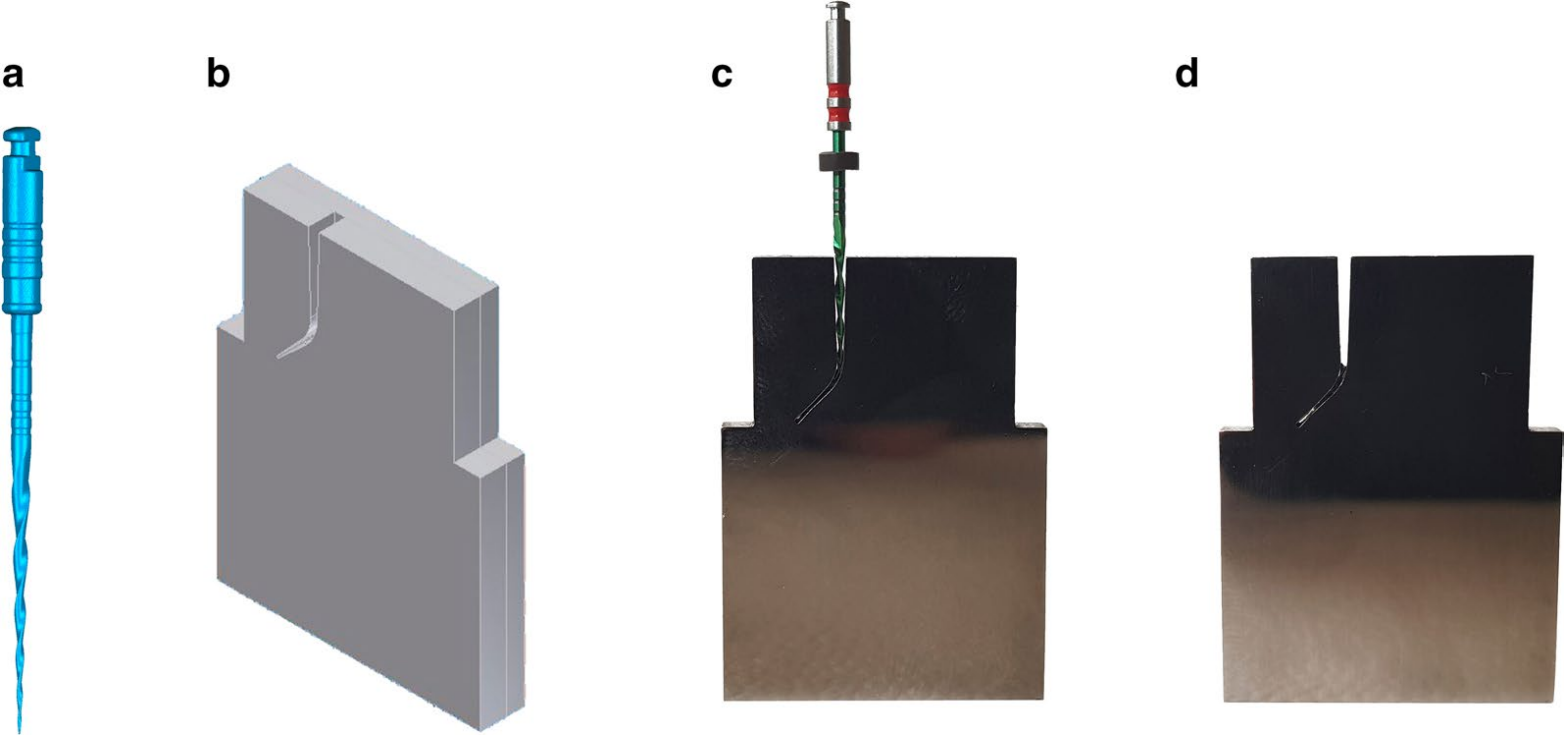
**a** STL file of the endodontic reciprocating file, **b** STL file of the artificial root canal, **c** adjustment of the endodontic reciprocating file with the artificial root canal walls and **d** fracture of the endodontic reciprocating file inside the artificial root canal

To obtain an accurate stereolithography (STL) file, endodontic reciprocating file (Procodile 25.06) underwent microcomputerized tomography scan (Skyscan 1176, Bruker-MicroCT, Kontich, Belgium) (Fig. 1a). STL file was used to design an anatomically based artificial root canal with a 60° curvature according to Schneider’s measuring technique [13] and 3 mm radius of curvature by inverse engineering technology using the CAD/CAE 2D/3D software (Fig. 1b). The artificial root canal was manufactured by electrical discharge machining (EDM) molybdenum wire-cut technology (Cocchiola S.A., Buenos Aires, Argentina). This process allowed intimate contact between endodontic reciprocating files and artificial root canal walls (Fig. 1c, d).

The endodontic reciprocating files randomly assigned to groups A and B were used with the ReFlex Dynamic and ReFlex Smart reciprocating movements respectively, and both were performed with a 6:1 reduction handpiece (EndoPilot® endodontic handpiece) and torque-controled motor (EndoPilot®, Schlumbohm, Brokstedt, Germany). It is not possible to provide torque and revolutions per minute (rpm), because handpiece management software adapts the reciprocating movement of files with regard to their resistance inside the artificial root canal, to reduce cyclic and torsional fatigue and, hence, to increase the fracture resistance. The handpiece management software continuously analyzes the resistance experienced by the reciprocating files inside the artificial root canal through an accurate mathematical algorithm. The Procodile files randomly assigned to group C were used by a 6:1 reduction handpiece, a torque-controlled motor (Silver Reciproc®; VDW, Munich, Germany) and Reciproc® reciprocating movement was performed by the Silver Reciproc endodontic handpiece according to the manufacturer’s instructions [14].

To allow accurate adjustment with their respective endodontic handpiece supports during the dynamic cyclic fatigue tests, both endodontic handpieces were scanned (3D Geomagic Capture Wrap, 3D Systems^©^, Rock Hill, SC, USA).

All endodontic reciprocating files were used in the dynamic cyclic fatigue device to a frequency of 60 pecking movements/min according to a previous study [12]. To reduce the friction between the reciprocating files and the artificial canal walls, special high-flow synthetic oil (Singer All-Purpose Oil; Singer Corp., Barcelona, Spain) designed for lubrication of mechanical parts was applied.

All files were used until fracture occurred and the time to failure, the number of cycles of in-and-out movements and the length of fractured files tip were measured and recorded.

### Statistical tests

SAS 9.4 (SAS Institute Inc., Cary, NC, USA) was used to statistical analysis of all variables. Descriptive statistics are expressed as mean and standard deviation (SD) for quantitative variables. By comparing the time to failure (seconds) and the number of pecking movements (cycles of in-and-out movements), comparative analysis was performed using ANOVA. In addition, Weibull characteristic strength and Weibull modulus were calculated and their 95% confidence interval for each group. The statistical significance was set at *p* < 0.05.

## Results

**Fig. 2:**
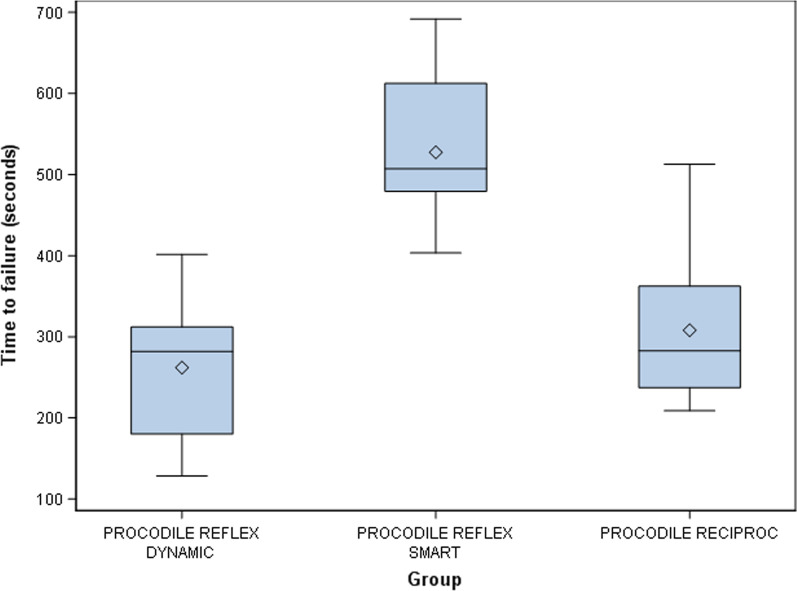
Box plots of time to failure of experimental groups. Horizontal line in each box represents median value

The means and SD values for time to failure (seconds) of the study groups are displayed in Table 1 and Fig. 2. The mean time to fracture of Procodile Reflex Dynamic was 261.95 s and SD 83.32, while that of Procodile Reflex Smart was 527.43 s and SD 89.31; the mean time to fracture for Procodile with the Reciproc movement was 308.07 s and SD 92.04.

**Table 1:** Descriptive statistics of time to failure

	*n*	Mean	SD	Minimum	Maximum	Fracture length
Procodile ReFlex Dynamic	10	261.95^a^	83.32	128.28	401.69	3.18
Procodile ReFlex Smart	10	527.43^b^	89.31	403.35	691.90	3.15
Procodile Reciproc	10	308.07^c^	92.04	208.82	512.92	3.18

^a,b,c^Statistically significant differences between groups (*p* < 0.05)

ANOVA revealed statistically significant differences between the time to failure of ReFlex Dynamic and Reciproc reciprocating movement (*p* < 0.001), and between ReFlex Smart and Reciproc reciprocating movement (*p* < 0.001). However, no statistically significant differences were observed between the time to failure of ReFlex Dynamic and ReFlex Smart reciprocating movement (*p* = 0.253).

**Fig. 3:**
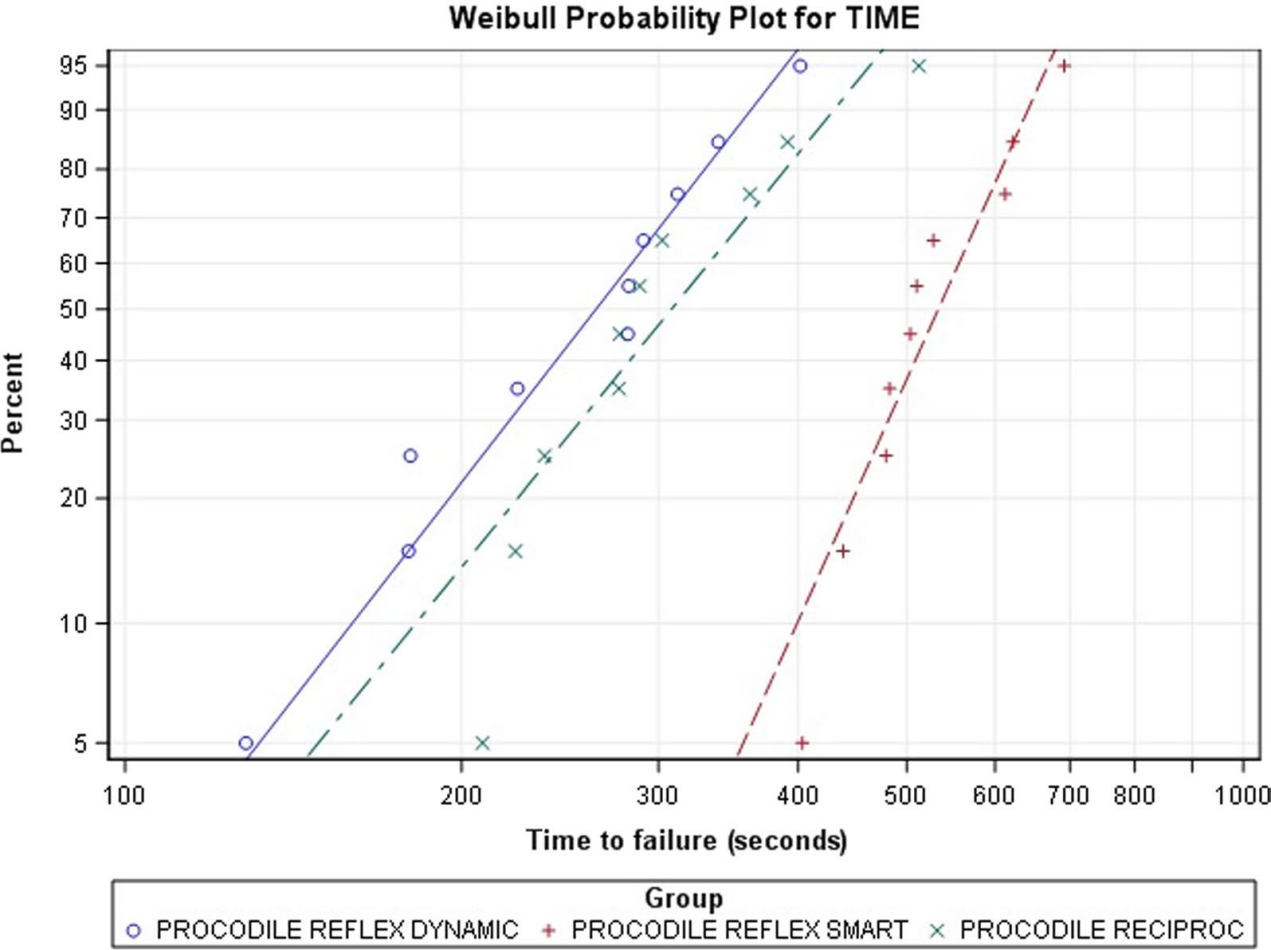
Weibull probability plot of time to failure

The scale distribution parameter (η) of Weibull statistics showed statistically significant differences between the time to failure of ReFlex Dynamic and Reciproc reciprocating movement (*p* < 0.001), and between ReFlex Smart and Reciproc reciprocating movement (*p* < 0.001). However, no statistically significant differences were observed between the time to failure of ReFlex Dynamic and ReFlex Smart reciprocating movement (*p* = 0.215) (Table 2 and Fig. 3). The shape distribution parameter (β) of Weibull statistics did not show statistically significant differences between the time to failure of ReFlex Dynamic and Reciproc reciprocating movement (*p* = 0.069), between ReFlex Smart and Reciproc reciprocating movement (*p* = 0.112), and between ReFlex Dynamic and ReFlex Smart reciprocating movement (*p* = 0.889). ReFlex Smart reciprocating movement presented the highest shape distribution parameter of Weibull statistics (6.4889), therefore the highest predictability compared to ReFlex Dynamic and Reciproc, which showed the lowest shape distribution parameter of Weibull statistics (3.5825) and the largest scatter of fracture point, hence the least predictable cyclic fatigue resistance behaviour (Table 2 and Fig. 3).

**Table 2:** Weibull statistics of time to failure

Weibull shape (β)					Weibull scale (η)			
	Estimate	SE	Lower	Upper	Estimate	SE	Lower	Upper
Procodile ReFlex Dynamic	37.555	0.9442	2.944	61.472	290.668	277.881	2.442.749	3.458.726
Procodile ReFlex Smart	64.889	15.332	40.837	103.108	5.645.218	292.099	5.100.788	6.247.758
Procodile Reciproc	35.825	0.8125	22.969	55.876	3.411.025	320.377	2.837.505	4.100.466

**Fig. 4:**
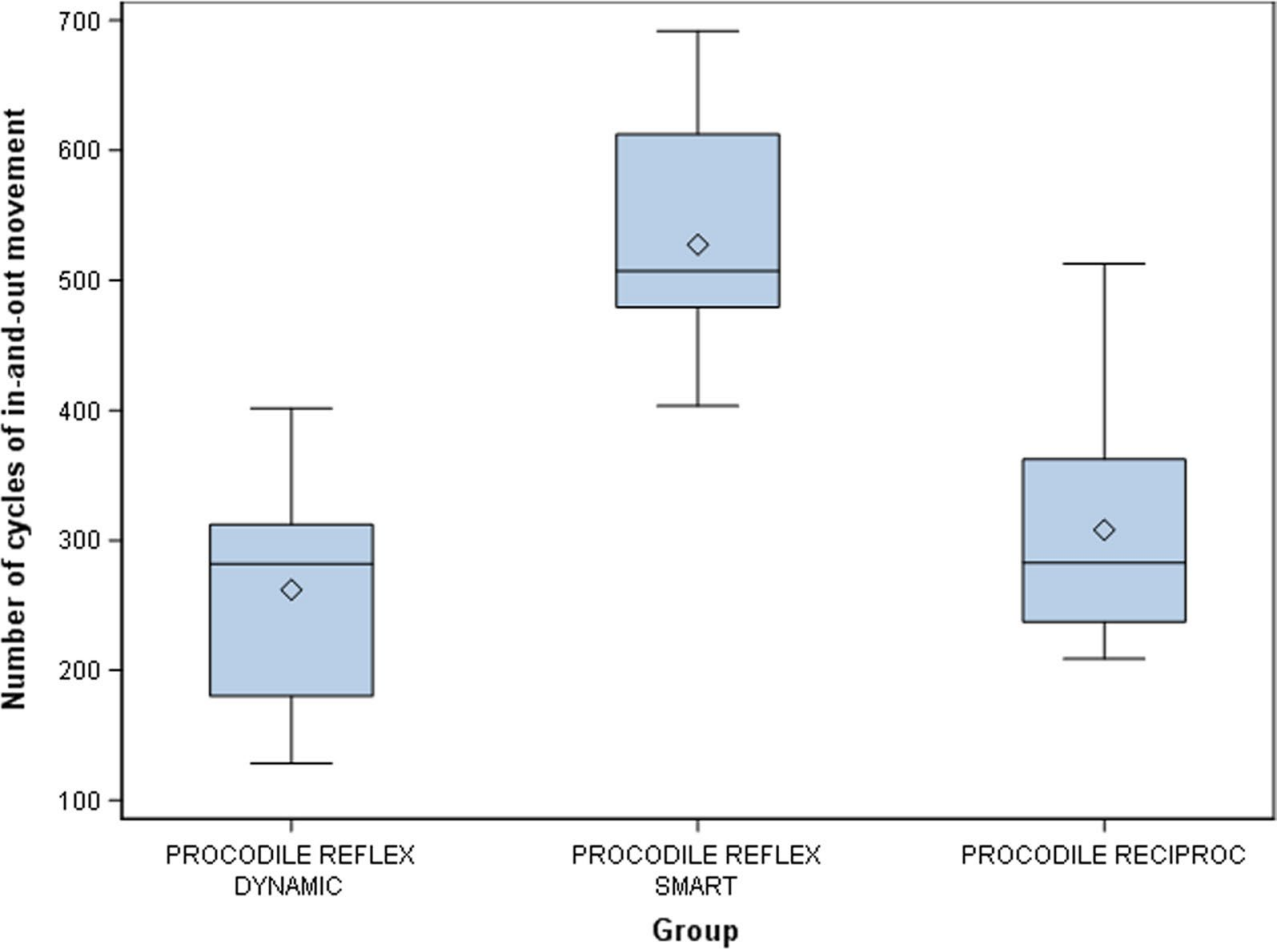
Box plots of number of cycles of in-and-out movement of experimental groups. Horizontal line in each box represents median value

The means and SD values for the number of cycles of in-and-out movement of the study groups are displayed in Table 3 and Fig. 4. The results for the number of cycles of in-and-out movement were the same as for the time to failure since a frequency of 60 pecking movements/min was used during for the process.

**Table 3:** Descriptive statistics of the number of cycles of in-and-out movement

	*n*	Mean	SD	Minimum	Maximum	Fracture length
Procodile ReFlex Dynamic	10	261.95^a^	83.32	128.28	401.69	3.18
Procodile ReFlex Smart	10	527.43^b^	89.31	403.35	691.90	3.15
Procodile Reciproc	10	308.07^c^	92.04	208.82	512.92	3.18

^a,b,c^Statistically significant differences between groups (*p* < 0.05)

ANOVA, in relation to the number of cycles of in-and-out movement, showed the same results as the time to failure, due to a frequency of 60 pecking movements/min was used during the dynamic cyclic fatigue tests.

**Fig. 5:**
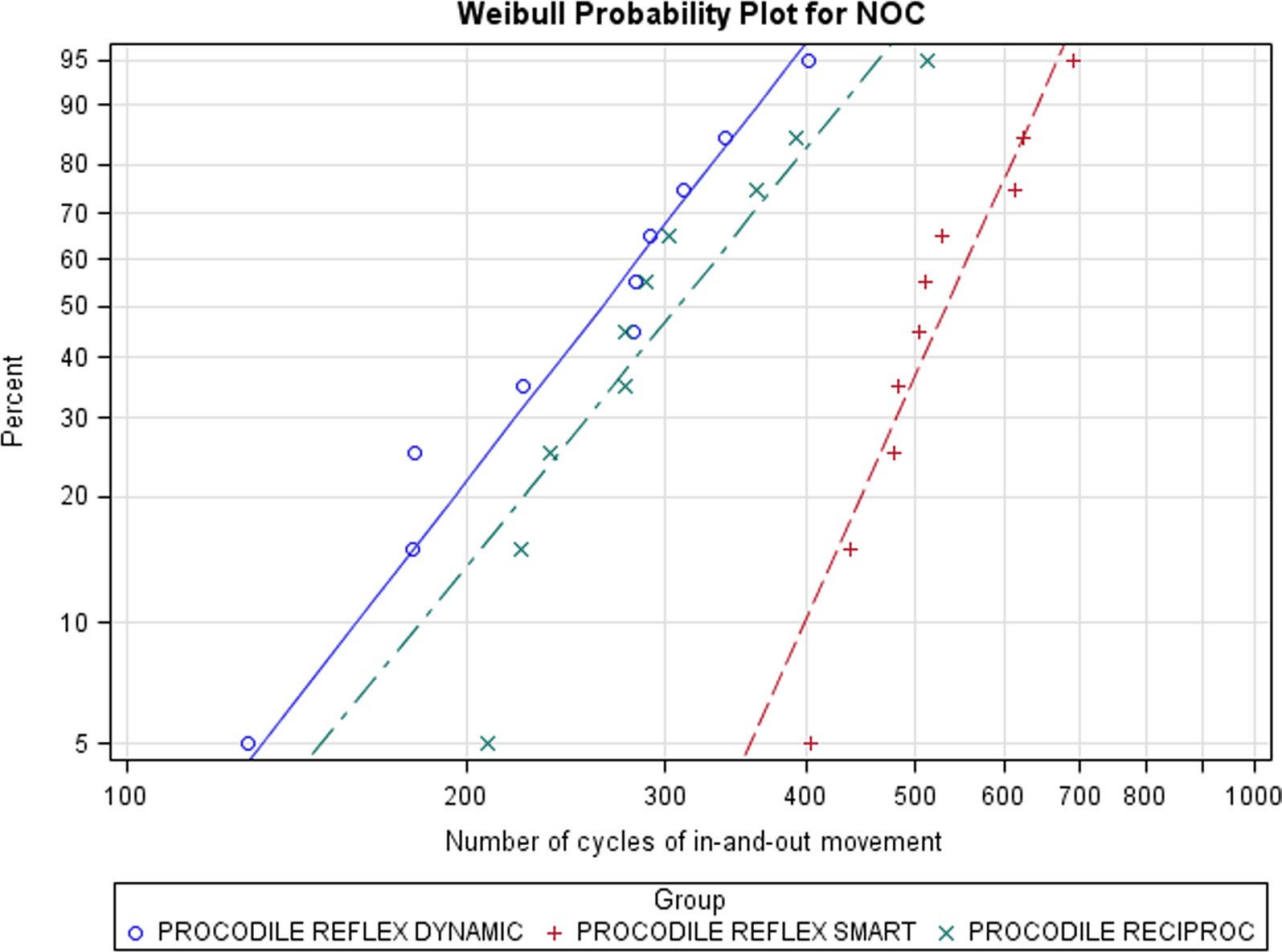
Weibull probability plot of number of cycles to failure (NOC)

The scale (η) and shape (β) distribution parameters of Weibull statistics related to the number of cycles of in-and-out movement also showed the same results as the time to failure because a frequency of 60 pecking movements/min was used during the dynamic cyclic fatigue tests (Table 4 and Fig. 5).

**Table 4:** Weibull statistics of number of cycles of in-and-out movement

Weibull shape (β)					Weibull scale (η)			
	Estimate	SE	Lower	Upper	Estimate	SE	Lower	Upper
Procodile ReFlex Dynamic	37.555	0.9442	2.944	61.472	290.668	277.881	2.442.749	3.458.726
Procodile ReFlex Smart	64.889	15.332	40.837	103.108	5.645.218	292.099	5.100.788	6.247.758
Procodile Reciproc	35.825	0.8125	22.969	55.876	3.411.025	320.377	2.837.505	4.100.466

The mean length of the fractured fragments was 3.18 mm in Procodile Reflex Dynamic and Procodile Reciproc, and 3.15 mm in Procodile Reflex Smart. These results were not statistically significantly different among all instruments tested (*p* > 0.05) (Tables 1, 3).

## Discussion

The results obtained in the present study reject the null hypothesis (H0), which states that there would be no difference between the effects of reciprocating movements on the cyclic fatigue resistance of endodontic rotary instruments.

When comparing cyclic fatigue resistance of reciprocating systems and conventional rotary systems, most studies have reported that reciprocating motion improves the cyclic fatigue resistance of endodontic instruments versus continuous rotation [15]. Cyclic fatigue has been tested in artificial canals using tubes, curved metal guiding slopes, plastic blocks and needles with curvatures; however, the artificial root canal should be custom-designed to ensure immediate contact with the endodontic file. In addition, only dynamic devices simulate the pecking movement performed by the operator, which represents the time during which the file remains in the canal. Olcay et al. [16] demonstrated that Wave One Gold endodontic reciprocating files showed a significant difference (*p* = 0.00) in time to failure (239.60 ± 12.84 s) compared to Protaper Next (161.40 ± 6.68 s) and 2Shape (77.73 ± 2.61 s) conventional rotary files; however, this was a static cyclic fatigue test with an artificial root canal with 1.5 mm wide parallel walls and 5 mm radius of curvature and different cross-sections and alloys. Scott et al. [17] analyzed the alloy influence on the cyclic fatigue resistance of reciprocating systems by comparing Wave One Primary, Wave One Gold Primary and EdgeFile X1, and concluded that endodontic reciprocating files manufactured with novel heat-treated alloys showed better cyclic fatigue resistance than those made with traditional M-Wire alloys; however, this was also a static cyclic fatigue test with an artificial root canal of parallel walls and 5 mm radius of curvature and different apical diameters. Al-Obaida et al. [18] also compared the cyclic fatigue resistance of five novel heat-treated manufactured NiTi reciprocating systems in canals with single and double curvature and demonstrated that Reciproc Blue (421.92 ± 155.09/251.25 ± 47.05 s) endodontic reciprocating files exhibited significantly (*p* < 0.05) higher cyclic fatigue resistance, followed by Reciproc (180.42 ± 35.43/160.58 ± 29.98 s) and Wave One Gold (167.67 ± 26.73/122.92 ± 26.54 s) for both types of artificial root canals. Regardless of the alloy of the reciprocating system, the cross-section that showed the best response to cyclic fatigue was the double-S cross-section of Reciproc and Reciproc Blue, which was the same as Procodile. Similarly, Sekar et al. [19] reported in their study that the cross-section that had the most influence on cyclic fatigue resistance was the double-S cross-section of Mtwo endodontic rotary files. Alsilani et al. [20] stated that the double-S cross-section of the Reciproc system presented a statistically significantly (*p* < 0.001) mean time to failure (301.13 ± 54.463/836.53 ± 67.960 s) compared to One Shape (187.73 ± 33.457/275.27 ± 58.410 s) and Revo-S SU (116.67 ± 37.663/197.60 ± 41.092 s) in both continuous rotation and reciprocating movement. Furthermore, Di Nardo et al. [21] compared the cyclic fatigue resistance of an NiTi and a novel heat-treated manufactured NiTi reciprocating system (Reziflow and Wave One Gold, respectively) and showed that the NiTi system presented significantly (*p* < 0.05) higher cyclic fatigue resistance (50.75 ± 20.06 s) than the heat-treated system (30.13 ± 9.40 s). Although this was also a static cyclic fatigue test with an artificial root canal of parallel walls and 90° and 5 mm radius of curvature, Reziflow has a similar design and manufacturing process as Procodile. These findings might suggest that the cross-section design of the endodontic files might also be a factor to consider in the results of cyclic fatigue tests above the manufacturing process. The longer time to failure results associated with the ReFlex Smart reciprocating movement (527.43 ± 89.31 s) can be attributed to the double reverse direction performed by EndoPilot endodontic handpiece, which allows reduced cyclic fatigue of the files. The dynamic cyclic fatigue test could also have an influence on the high results obtained compared to studies of static cyclic fatigue tests. Keleş et al. [22] reported that cyclic fatigue resistance was significantly (*p* < 0.05) greater in dynamic than static cyclic fatigue tests at room temperature for four endodontic reciprocating files—Wave One (177.9 ± 46.9/106.2 ± 36.6 s), Wave One Gold (258.9 ± 47.9/175.3 ± 60.3 s), Reciproc (292.4 ± 81.3/196.7 ± 55.6 s), and Reciproc Blue (275.9 ± 86.4/214.4 ± 108.4 s)—and provided a better simulation of the clinical environment, because compression and tensile stresses are distributed over a wider area along the file surface [23].

In this study, the time to failure and the number of cycles of in-and-out movement were analyzed to determine the influence of novel reciprocating movement on the cyclic fatigue resistance of NiTi endodontic reciprocating files; however, it was not possible to calculate the number of cycles to fracture in groups A and B because the rpm could vary during the tests by EndoPilot handpiece management software. This has been considered a limitation of the present study and highlights the need for an international standard for testing the cyclic fatigue resistance of NiTi endodontic reciprocating files, because several self-designed devices and methods have been used with different results [24]. However, none of these custom-made devices have been capable of dynamically testing the cyclic fatigue of NiTi endodontic reciprocating files in vitro with an automatic detection system and an anatomically based artificial root canal. We have not found comparative studies on the resistance to fracture of the Procodile files and the Smart and Reflex movements to be able to discuss our results.

An angle of curvature of 60° was selected to design the artificial root canal, because Topçuoğlu et al. [25] reported that artificial root canals with a 45° angle of curvature did not exhibit significant (*p* ˃ 0.05) differences between the cyclic fatigue resistance of R-Pilot (394.5 ± 45.3 s) and WaveOne Gold (412.4 ± 55.2 s) glider files; however, artificial root canals with 60° angle of curvature showed that WaveOne Gold (368.3 ± 44.1 s) had significantly (*p* < 0.05) greater cyclic fatigue resistance than R-Pilot (247.2 ± 36.2 s).

Although there are differences between reciprocating motions (speed and angle), further studies are needed to determine the most favorable motions for root canal treatment. Iacono et al. [26] analyzed the influence of reciprocating motion and reciprocating system alloys on cyclic fatigue resistance and stated that the experimental movement with different rotation angles, based on sinusoidal acceleration, showed a positive impact on the cyclic fatigue resistance of reciprocating instruments. The different values of CW and CCW reciprocating angles between ReFlex and the number of cycles of reciprocation per second could explain the statistical differences between the cyclic fatigue resistance of reciprocating movements. The CW and CCW reciprocating angles are specific for the endodontic reciprocating systems, and the CCW reciprocating angle should be smaller than the elastic limit of each system material. Ha et al. [27] reported a distortion angle and torsional load at the pseudo-elastic limit for the Reciproc system of 214 ± 25° and 1.78 ± 0.18 Ncm, respectively. However, the Reciproc Blue system showed a distortion angle and torsional load at the pseudo-elastic limit of 253 ± 19° and 1.57 ± 0.21 Ncm, respectively. The distortion angle of both systems is greater than the CCW reciprocating angle (150°), keeping the instrument below its pseudo-elastic limit [28] while extending its lifespan and clinical efficiency. In addition, it is probable that the angular speed (ω) of the angular displacement (θ) (mainly at the CCW angle) could increase the metal fatigue of the files, although it remains a concern [29]. It is known that the Reciproc reciprocating movements perform a θ of 2617 rad (150°) and a ω of 31.415 rad/s (300 rpm) at the CCW angle [11]; however, Wave One performs a θ of 2967 rad (170°) and a ω of 36.651 rad/s (350 rpm) [10, 11]. It is probable that the higher θ and ω values of Wave One compared to Reciproc and the higher cutting surface contact of Wave One (convex triangular cross-section) compared to Reciproc and Procodile (double-S cross-section) could also affect the cyclic fatigue resistance of these reciprocating instruments. Procodile also presents a double-S cross-section, but unlike the others, it is made of conventional NiTi alloy; however, its variable internal taper offers greater flexibility.

The conclusion derived from this study is that novel reciprocating movements increase the cyclic fatigue resistance of endodontic reciprocating files compared with traditional reciprocating movements. Nevertheless, further research are needed to determine the influence of these novel reciprocating movements on the cyclic fatigue resistance of heat-treated manufactured NiTi reciprocating systems.

## Conclusions

In conclusion, within the limitations of this study, our results showed that ReFlex Smart reciprocating movement increased the cyclic fatigue resistance of endodontic reciprocating files compared with ReFlex Dynamic and Reciproc reciprocating movements.

## Data Availability

The datasets used and/or analysed during the current study are available from the corresponding author on reasonable request.

## Abbreviations

CAD/CAE: Computer-aided design/computer-aided engineering.
CCW: Counterclockwise
CW: Clockwise
EDM: Electrical discharge machining
LED/LDR: Light-emitting diode/light-dependent resistor
NOC: Cycles to failure
NiTi: Nickel–titanium
RPM: Revolutions per minute
SD: Standard deviation
STL: Standard Triangle Language
